# Exploring the Potential of s-Triazine Derivatives as Novel Antifungal Agents: A Review

**DOI:** 10.3390/ph18050690

**Published:** 2025-05-07

**Authors:** Haoyan Liao, Menglu Liu, Mengyuan Wang, Dazhi Zhang, Yumeng Hao, Fei Xie

**Affiliations:** 1 Student Bridge, College of Basic Medical Sciences, Naval Medical University, No. 800 Xiangyin Road, Shanghai 200433, China; 2 Department of Organic Chemistry, School of Pharmacy, Naval Medical University, No. 325 Guohe Road, Shanghai 200433, China

**Keywords:** heterocyclic compound, s-triazine derivatives, antifungal activity, minimum inhibitory concentration

## Abstract

The growing incidence and prevalence of invasive fungal infections (IFIs) and the emergence of antimicrobial resistance compound clinical antifungal therapies. Given the significant threat posed by IFIs and the limits of the current antifungal agents, the search for novel, effective therapeutic options remains a compelling area of antifungal drug discovery. The s-triazine (1,3,5-triazine) scaffold, renowned for its structural versatility, ease of functionalization, and diverse biological profiles, has been extensively studied in medical chemistry. Driven by this privileged structure, several s-triazine derivatives have been synthesized through molecular hybridization and screened for their antifungal activities. Some of them demonstrated potent efficacy against pathogenic fungi, including *Candida*, *Cryptococcus*, and *Aspergillus* species. Structure–activity relationship (SAR) studies are also discussed whenever possible, underlying the essential substituents for their antifungal effect. This review provides a summary of recent advancements (2014–2024) in the development of antifungal agents featuring the s-triazine scaffold and highlights the antifungal activity of s-triazine derivatives, aiming to prompt further progress in this field.

## 1. Introduction

The global incidence of life-threatening invasive fungal infections (IFIs) has increased significantly. It is estimated that over 6.5 million people have a life-threatening fungal infection worldwide, with approximately 2.5 million deaths each year [1,2], posing a significant impact on public health. Moreover, the incidence and prevalence of IFIs continue to be exacerbated by acquired immunodeficiency syndrome (AIDS), influenza, more recently by the COVID-19 outbreaks, and the emergence of multidrug resistance fungi [3,4]. Given the threat of fungal infection, the World Health Organization developed a fungal priority pathogens list (WHO FPPL, 2022) to help galvanize global action, which classified four fungal pathogens (*Cryptococcus neoformans*, *Candida auris*, *Aspergillus fumigatus*, and *Candida albicans*) as the “critical” group and a further fifteen fungal pathogens (including *Candida glabrata*, *Candida parapsilosis*, *Candida krusei*, *Fusarium* spp., and the *Mucorales*) as medium or high priority group [5]. *Candida*, *Cryptococcus*, and *Aspergillus* species are the primary opportunistic fungal pathogens, accounting for nearly 90% of deaths in IFIs [6]. *Candida auris* is characterized by a high level of multidrug resistance, with mortality rates as high as 72%, and has become an urgent healthcare threat [7].

Treatment of IFIs is highly challenging. Currently, only a few types of antifungal drugs are used in the clinic to treat IFIs: azoles (fluconazole, itraconazole, voriconazole, posaconazole, isavuconazole, and oteseconazole), echinocandins (caspofungin, micafungin, and anidulafungin), polyene antibiotics (amphotericin B), antimetabolites (5-fluorocytosine), and triterpenoids (ibrexafungerp). However, issues with existing antifungal drugs include relatively narrow spectrums of activity, multiple and diverse drug–drug interactions, limitations to access worldwide, frequent acquired and innate drug resistance, etc. [8,9]. Therefore, there is an urgent need to develop new antifungal agents with novel chemical scaffolds.

**Figure 1:**
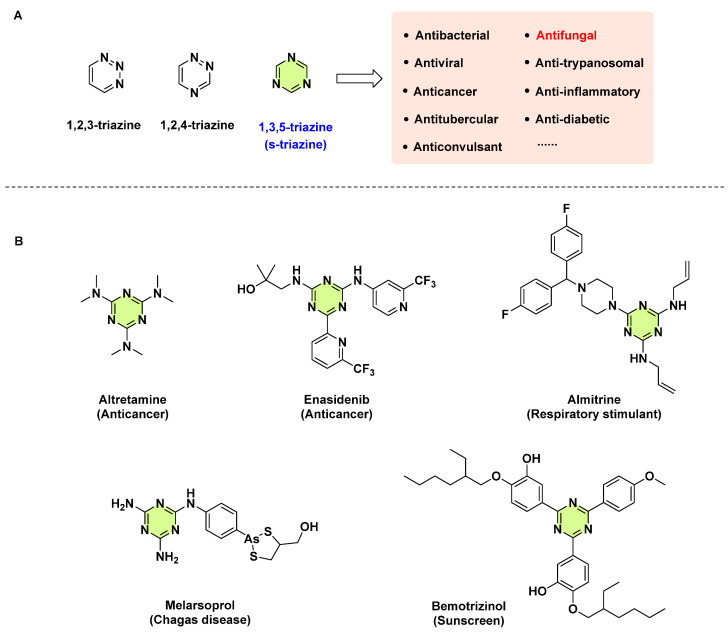
(**A**) Three isomers of triazine and the wide range pharmacological activities of s-triazine. (**B**) Some commercial drugs containing the s-triazine ring.

The triazine ring is one of the most essential heterocyclic, pharmacologically active moieties in drug molecules. The planar, six-membered ring of triazine exists in three isomeric forms depending on the position of the nitrogen atom, namely 1,2,3-triazine, 1,2,4-triazine, and 1,3,5-triazine, known as s-triazine. Of these three isomers, the rigid symmetrical structure of s-triazine has received much attention in medicinal chemistry. It possesses diverse biological profiles such as antibacterial [10], antiviral [11], anticancer [12], antitubercular [13], anticonvulsant [14], etc. (Figure 1A). Moreover, many approved drugs available on the market (e.g., altretamine, enasidenib, almitrine, melarsoprol, and bemotrizinol) have an s-triazine moiety (Figure 1B), indicating its favorable safety and pharmacokinetic properties. Apart from their application in medicinal chemistry, s-triazine derivatives are also used as herbicides, insecticides, corrosion inhibitors, energetics, and new materials [15,16,17,18].

**Figure 2:**
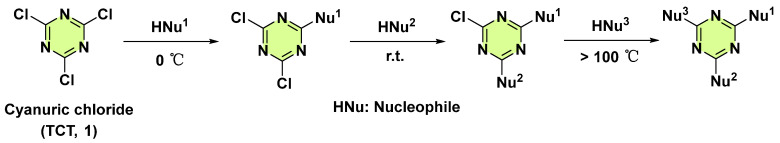
General synthesis routine of a substituted s-triazine from cyanuric chloride.

In addition, s-triazine is an ideal framework for constructing novel drug candidates due to the ease of synthesizing the s-triazine core from simple starting materials or the availability of cyanuric chloride or 2,4,6-trichloro-1,3,5-triazine (TCT, **1**), alongside the ability to explore the chemical space within the core. Figure 2 presents a temperature-dependent selective replacement of the chlorine atoms in **1** with sequential nucleophilic substitutions (typically N-, O-, S- or P-nucleophiles) that allow the extensive preparation of mono-, di-, and tri-substituted s-triazine derivatives. The replacement of the first Cl atom was performed at 0 °C in the presence of a base such as Na_2_CO_3_, NaHCO_3_, triethylamine, diisopropylethylamine, etc., while the second one needs room temperature, and the third heating or reflux.

Although many reviews have reported the synthesis, structure–activity relationships (SARs) and the biological application of triazine derivatives, few of them focus on the antifungal properties of s-triazine compounds [19,20,21,22,23,24]. Given the grave need to develop new therapeutic options for the treatment of IFIs, we cover this review by highlighting recent studies of s-triazine derivatives as potential antifungal agents from publications between 2014–2024. This review not only attracts the attention of medicinal chemists but also provides insight into the antifungal drug development of s-triazine derivatives in future work.

## 2. Methodology

A systematic literature search was performed in PubMed, SciFinder, Scopus, and Web of Science. A search on s-triazine derivatives with antifungal activity was conducted by using combinations of keywords, including “triazines”, “s-triazine”, “1,3,5-triazine”, “derivatives”, “compounds”, “agents”, “antimicrobial activity”, “antifungal activity”, “antifungal”, “fungi”, and “fungal”. Then, the literature was saved after being imported into Endnote, and duplicates were removed. Next, the literature was checked according to the following inclusion and exclusion criteria.

(a) Inclusion: the literature (1) published between 2014 and 2024, (2) available in English or with an English abstract if published in another language, and (3) studies on pathogenic fungi.
(b) Exclusion: the literature on (1) purely computational, structural, or in silico studies or (2) without full-text availability.

## 3. Antifungal Activities of s-Triazine Based Derivatives

**Figure 3:**
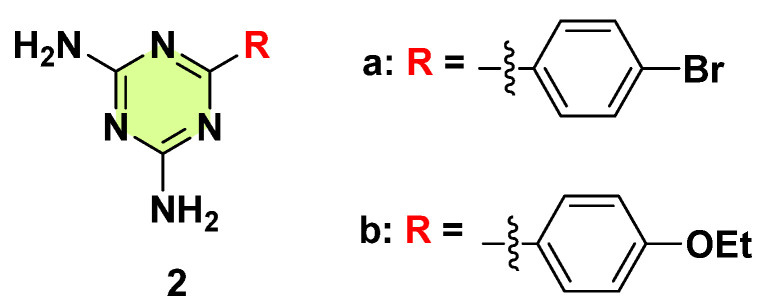
Chemical structures of the s-triazine derivatives **2a**,**b**.

In 2020, Patil et al. reported the synthesis of a series of s-triazine derivatives through a one-step reaction by mixing 2-cyanoguanidine with various substituted benzonitriles to yield fifteen 1,3,5-triazine-2,4-diamines derivatives (Figure 3) [25]. The antifungal activity of each compound was examined against two fungal strains: *C. albicans* and *C. neoformans*. Compound **2a**, bearing a 4-Br substituted phenyl group, displayed moderate fungal growth inhibition (~25% at 32 µg/mL) against both fungi. Compound **2b**, bearing a 4-ethyl substituted phenyl, showed the highest growth inhibition (~30% at 32 µg/mL) against *C. neoformans*. The SAR suggested that either electron-withdrawing or electron-donating group substitution on the aryl did not play any role in the antifungal profiles.

**Figure 4:**
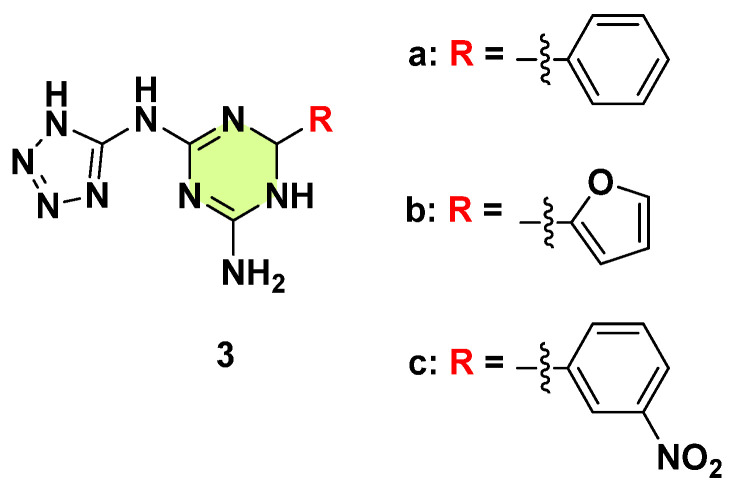
Chemical structures of the s-triazine derivatives **3a**–**c**.

Mekheimer et al. (2022) synthesized and reported various *N*^2^-(tetrazol-5-yl)-6-substituted-5,6-dihydro-1,3,5-triazine-2,4-diamines through the microwave reaction of 5-amino-1,2,3,4-tetrazole, cyanamide, and aromatic or heteroaromatic aldehydes (Figure 4) [26]. These s-triazine-tetrazole analogs were subsequently screened for in vitro antimicrobial activity. Notably, compounds **3a**–**c** demonstrated excellent antifungal efficacy against *C. albicans* with minimum inhibitory concentration (MIC) values of 1.475 × 10^−8^, 1.288 × 10^−3^, and 2.1851 × 10^−4^ µg/mL, respectively, which was significantly more efficacious than the reference fluconazole (MIC: 0.857 µg/mL). The replacement of the phenyl group with other aryl groups or a substitution on the benzene ring resulted in a loss of antifungal activity. Furthermore, compounds **3a**–**c** exhibited good inhibition of *Candida* 14α-demethylase enzyme, with IC_50_ values of 7.451 ± 0.404, 25.066 ± 1.358, and 3.369 ± 0.183 µg/mL, respectively, as determined by a rapid fluorescence-based screening method. Molecular docking studies indicated that compounds **3a**–**c** demonstrated good binding affinity to the human CYP51 protein (PDB code: 3LD6) and possessed acceptable ADME properties. Notably, effective antifungal drugs require fungal selectivity to avoid unwanted side effects. While inhibition of fungal CYP51 can be effective, inhibiting human CYP51 could generate toxicity. Therefore, it is necessary to consider the safety of these compounds in regard to mammalian cells.

**Figure 5:**
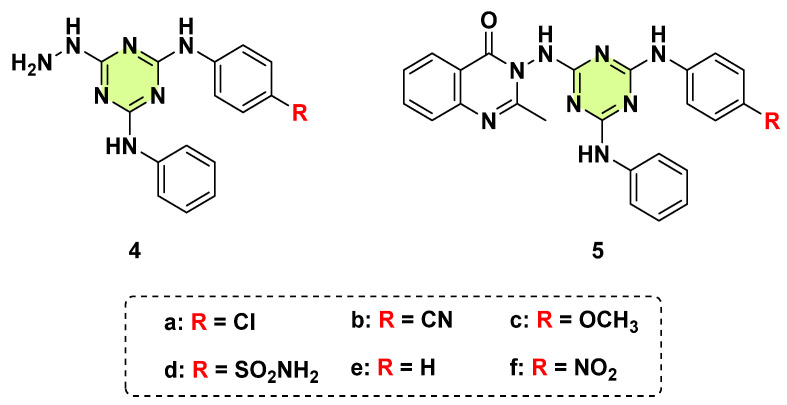
Chemical structures of the s-triazine derivatives **4a**–**f** and **5a**–**f**.

Hybridization of different bioactive moieties into a single molecule have the potential to improve the efficacy, reduce toxicity, enhance the pharmacokinetic properties, and overcome drug resistance [27]. Dinari et al. in 2018 reported a series of s-triazine-quinazolinone hybrids (Figure 5) [28]. The synthesized compounds were screened for their in vitro antimicrobial activities. The trisubstituted s-triazine hydrazine intermediates **4a**–**f** displayed moderate to weak inhibitory activity against *C. albicans*, with MICs ranging from 128 to 512 μg/mL. When a benzoxazinone moiety was added into the intermediates to afford target hybrids, **5a**–**f**, their anti-*C. albicans* activity improved 1- to 2-fold. In general, the antifungal activity of s-triazine-quinazolinone hybrids was poor and substantially inferior to their antibacterial activity.

**Figure 6:**
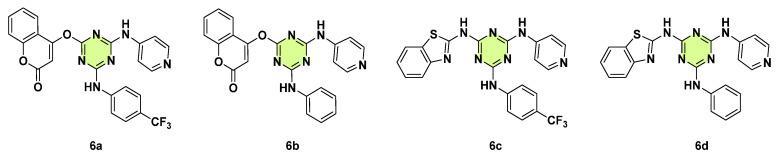
Chemical structures of the s-triazine derivatives **6a**–**d**.

In 2023, Zala et al. synthesized twelve molecular hybrids of s-triazine with coumarin and s-triazine with benzothiazole (Figure 6) [29]. These compounds were evaluated for their in vitro antifungal activities against *Trichoderma rubrum* and *C. albicans*. The hybrid **6a** (MIC: 100 μg/mL) was most effective against the *T. rubrum* strain and comparable to reference griseofulvin, whilst the remaining compounds had moderate activity, with MICs ranging from 500–1000 μg/mL. Compounds **6b**–**d** gave MIC values of 250 μg/mL against the *C. albicans* strain, exhibiting a potency 2-fold greater than griseofulvin.

**Figure 7:**
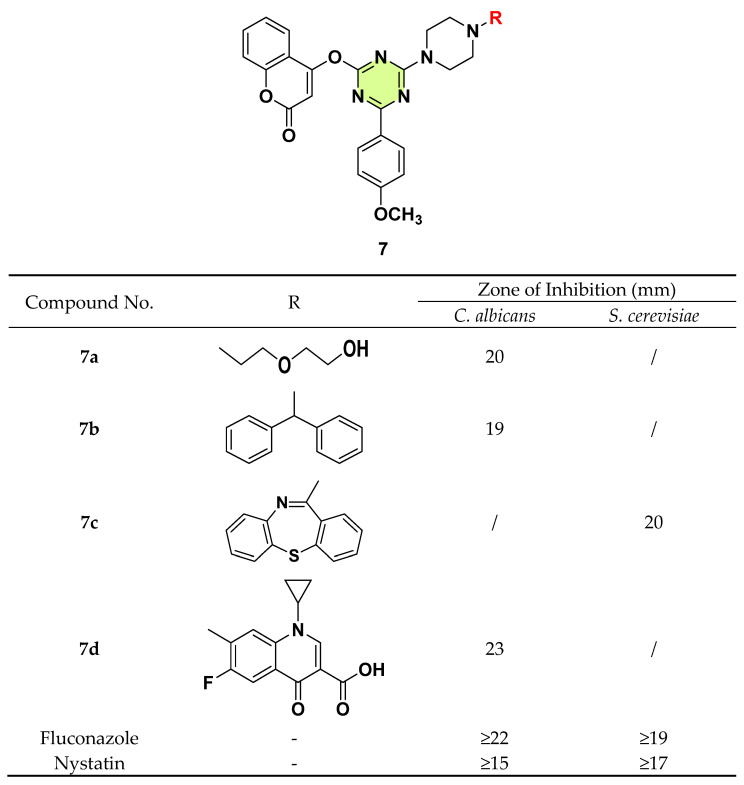
Chemical structures and antifungal activities of the s-triazine derivatives **7a**–**d**.

Sweta et al. (2014) synthesized clubbed coumarin and *N*-substituted piperazine s-triazine hybrids and tested their in vitro antifungal activity against *C. albicans* and *Saccharomyces cerevisiae* using the Kirby–Bauer disc diffusion method (Figure 7) [30]. The biological screening results indicated that compounds **7a** (inhibition zone: 20 mm), **7b** (inhibition zone: 19 mm), and **7d** (inhibition zone: 24 mm) displayed considerable anti-*C. albicans* activities, which were comparable to fluconazole and nystatin. Compound **7c**, bearing dibenzo [b,f]-thiazapine piperazine, showed a high inhibition effect against *S. cerevisiae*.

**Figure 8:**
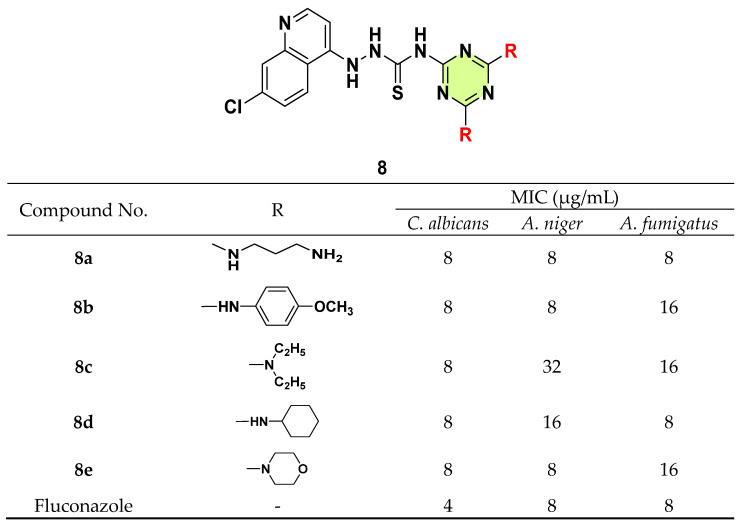
Chemical structures and antifungal activities of the s-triazine derivatives **8a**–**e**.

By fusion of triazine with other pharmacophoric fragments, Bhat et al. in 2019 prepared a series of 4-aminoquinoline-s-triazine derivatives (Figure 8) [31]. Compounds **8a**–**e** were the most potent in this series against *C. albicans* with MIC = 8 μg/mL. For *Aspergillus niger* and *A. fumigatus* strains, all the tested compounds showed moderate activity with MICs ranging from 8–32 μg/mL.

**Figure 9:**
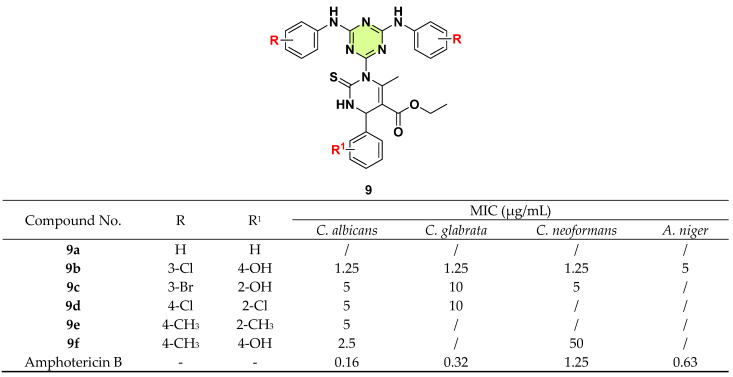
Chemical structures and antifungal activities of the s-triazine derivatives **9a**–**f**.

In 2020, Masih et al. synthesized a series of s-triazine-dihydropyrimidine hybrids (Figure 9) [32]. All synthesized compounds were evaluated for their in vitro antifungal activities against *C. albicans*, *C. glabrata*, *C. neoformans*, and *Aspergillus niger*. These compounds exhibited mild to moderate antifungal activity against the four tested strains. Notably, most compounds demonstrated better inhibition of *Candida* spp. than *C. neoformans* and *A. niger*. Compared with the unsubstituted analogs, introduction of substituents at R/R^1^ position could enhance the antifungal activity. For instance, compound **9a** exhibited no antifungal effect, whereas compound **9b** displayed the best and broad-spectrum activity against the tested strains with an MIC of 1.25–5 µg/mL. Compounds **9c**–**f** showed promising antifungal activity against *C. albicans* (MIC: 2.5–5 μg/mL). However, no clear SARs between the R and R^1^ substituents were observed.

**Figure 10:**
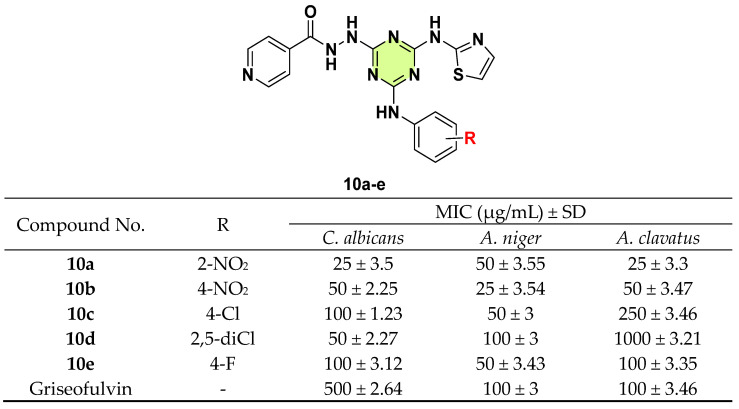
Chemical structures and antifungal activities of the s-triazine derivatives **10a**–**e**.

Desai et al. (2016) reported multiple s-triazine based thiazole hybrids [33]. The antifungal activities of the synthesized compounds were investigated in *C. albicans*, *A. niger*, and *A. clavatus* (Figure 10). The -NO_2_ substituted aniline was determined as essential to increase pharmacological activity. For example, compounds **10a** and **10b** showed broad and excellent inhibition against three tested fungi. Compounds **10c** and **10d** possessed potent inhibition against *C. albicans* and *A. niger*, whose MIC values were lower or equal to that of griseofulvin.

**Figure 11:**
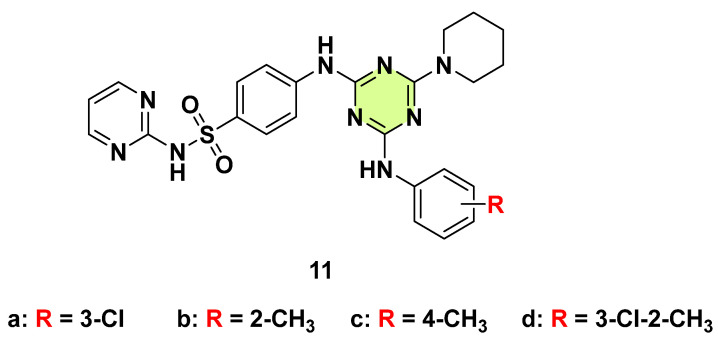
Chemical structures of the s-triazine derivatives **11a**–**d**.

A series of s-triazine-benzenesulfonamide hybrids were also evaluated for their antifungal activities against *C. albicans*, *A. niger*, and *Aspergillus clavatus* by Desai et al. in the year 2016 (Figure 11) [34]. All hybrids displayed better inhibitory activity against *C. albicans* than *A. niger* and *A. clavatus*. Among them, compounds **11a**–**d** demonstrated mild antifungal activity (MIC: 250 μg/mL) against *C. albicans*, while the remaining compounds showed weak (MIC: 500–1000 μg/mL) or no activity against *C. albicans*. Additionally, **11b** was identified as the most effective agent (MIC: 250 μg/mL) against *A. niger*, whereas it had no activity against *A. clavatus*. The SAR studies revealed that the antifungal activity of these s-triazine hybrids was significantly influenced by different R-substituents on the phenyl ring.

**Figure 12:**
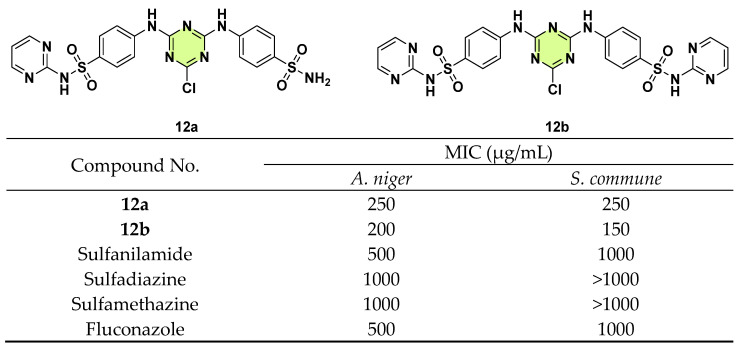
Chemical structures and antifungal activities of the s-triazine derivatives **12a**,**b**.

Similarly, s-triazine-bis-benzenesulfonamide hybrids 4-((4-chloro-6-((4-sulfamoylphenyl)amino)-1,3,5-triazin-2-yl)amino)-*N*-(pyrimidin-2-yl)benzenesulfonamide and 4,4′-((6-chloro-1,3,5-triazine-2,4-diyl)bis(azanediyl))bis(*N*-(pyrimidin-2-yl)benzenesulfonamide) were evaluated for in vitro antimicrobial activities against four bacterial strains and two fungal strains, *A. niger* and *Schizophyllum commune*, by Noureen and co-workers in 2022 (Figure 12) [35]. Compound **12a** (inhibition zone: 20 ± 0.51 mm against *A. niger*) is more potent than **12b** and fluconazole. In studies against *S. commune*, **12a** (inhibition zone: 22 ± 0.65 mm) and **12b** (inhibition zone: 25 ± 0.72 mm) displayed higher potency than sulfanilamide, sulfadiazine, sulfamethazine, and fluconazole. Moreover, the MIC values of the two compounds confirmed their antifungal activity. Cytotoxic studies indicated that compounds **12a** and **12b** had low hemolysis, suggesting a good safety profile.

**Figure 13:**
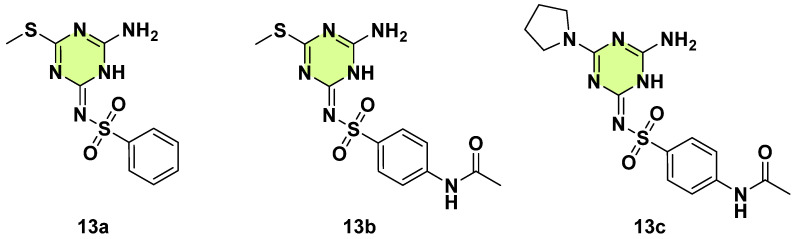
Chemical structures of the s-triazine derivatives **13a**–**c**.

In 2024, Mohamed-Ezzat et al. evaluated the potential of s-triazine sulfonamide conjugate as anti-microbial, antitumor, and anti-SARS-CoV-2 agents (Figure 13) [36]. Compounds **13a**–**c** were the most active compounds against *C. albicans*, showing a zone of fungal inhibition with the values 12.3 ± 0.6 mm, 13.3 ± 0.6 mm, and 9.6 ± 0.6 mm, respectively. It is worth noting that replacement of pyrrolidine with piperidine or morpholine led to loss of anti-*C. albicans* potency. Additionally, **13a** also demonstrated remarkable antiproliferative and antiviral potency.

**Figure 14:**
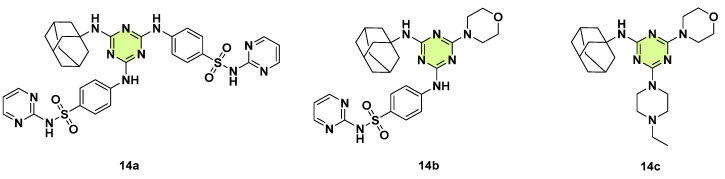
Chemical structures of the s-triazine derivatives **14a**–**c**.

In the same year, Kumawat et al. integrated multiple bioactive moieties such as adamantylamine, sulfamerazine, sulfadiazine, morpholine, thiazole, and piperazine into the s-triazine core (Figure 14) [37]. In vitro antifungal activity of these s-triazine hybrids was evaluated against *Malassezia furfur*. A total of 6 of 11 tested compounds revealed higher potency than ketoconazole. Notably, compound **14a** exhibited the highest activity against *M. furfur* (MIC: 8.13 ± 0.27 µg/mL), followed by **14b** (MIC: 9.34 ± 0.24 µg/mL) and **14c** (MIC: 12.21 ± 0.25 µg/mL). Furthermore, **14a** exhibited the highest antibacterial activity against *Pseudomonas chlororaphis*. In silico pharmacokinetic and ADME-T analysis of compounds **14a** and **14b** revealed favorable drug-like properties.

**Figure 15:**
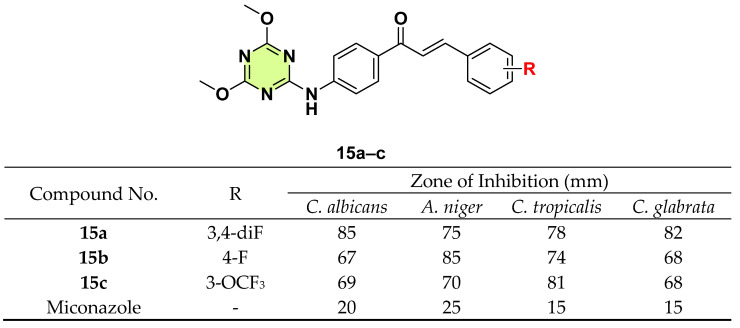
Chemical structures and antifungal activities of the s-triazine derivatives **15a**–**c**.

In another study, Shinde et al. (2021) synthesized a series of 4,6-dimethoxy-1,3,5-triazine and chalcone hybrids [38]. Antifungal activity testing was performed using four fungal strains (*C. albicans*, *A. niger*, *Candida tropicalis*, and *C. glabrata*) (Figure 15). Compound **15a** demonstrated the highest activity against *C. albicans* (inhibition zone: 85 mm) and *C. glabrata* (inhibition zone: 82 mm), while **15b** (inhibition zone: 85 mm) and **15c** (inhibition zone: 81 mm) showed excellent antifungal activity, especially against *A. niger* and *C. tropicalis*, respectively. Generally, a fluorine substituent on the benzene ring of chalcones was more effective for antifungal activity.

**Figure 16:**
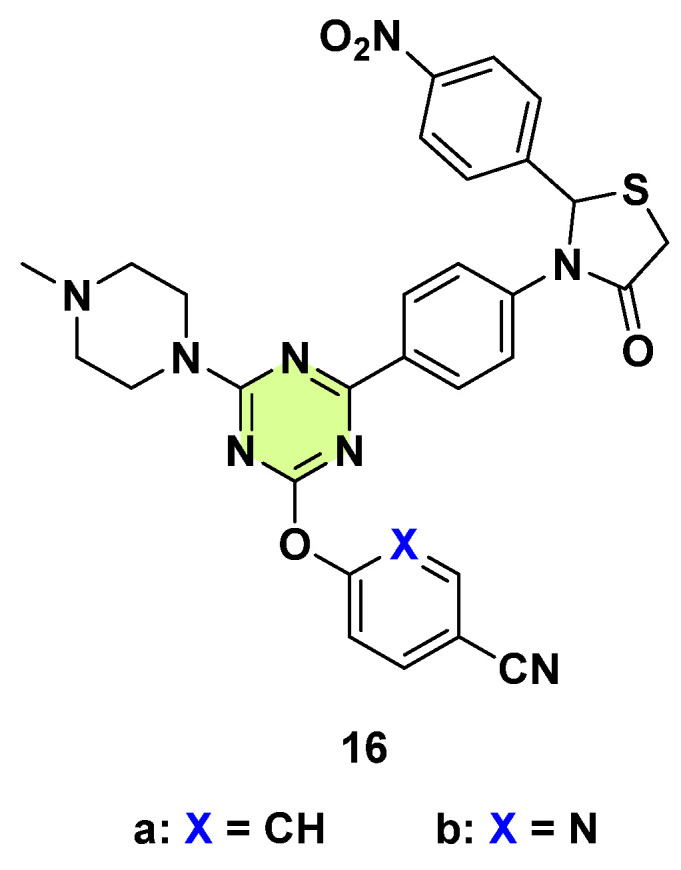
Chemical structures of the s-triazine derivatives **16a**,**b**.

Patel et al. in 2014 synthesized new thiazolidin-4-one fused s-triazine hybrids as potential antimicrobial and anticancer agents (Figure 16) [39]. The most active compounds, **16a** and **16b**, exhibited considerable activities (MIC: 3.12–25 μg/mL) against *A. niger* and *C. albican*, but they were less active than ketoconazole (MIC: 1.56 μg/mL). SAR studies suggested that both benzonitrile and nicotinonitrile were beneficial to increase the corresponding pharmacological activities.

**Figure 17:**
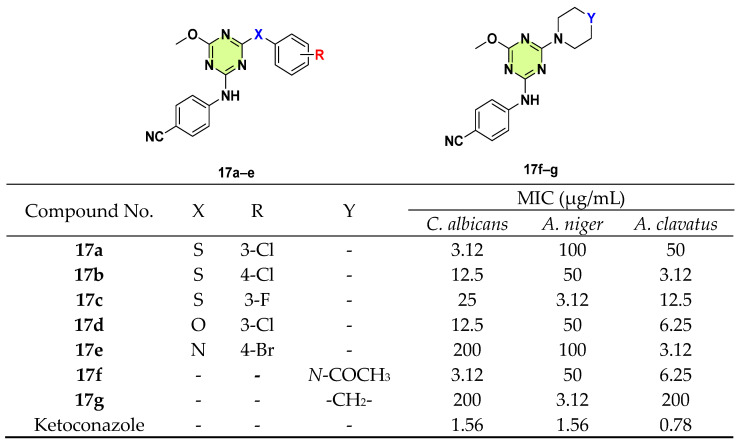
Chemical structures and antifungal activities of the s-triazine derivatives **17a**–**g**.

Mewada et al. in 2018, developed four classes of s-triazine based derivatives that incorporated the methoxy, 4-aminobenzonitrile moieties with phenol, thiophenol, aniline, and piperazine/piperidine/morpholine to a triazine nucleus (Figure 17) [40]. Compounds **17a** (MIC: 3.12 μg/mL against *C. albicans*), **17b** (MIC: 3.12 μg/mL against *A. clavatus*), **17c** (MIC: 3.12 μg/mL against *A. niger*), **17e** (MIC: 3.12 μg/mL against *A. clavatus*), **17f** (MIC: 3.12 μg/mL against *C. albicans*), and **17g** (MIC: 3.12 μg/mL against *A. niger*) showed the best inhibition activities, respectively. The 3-Cl substituted phenol derivative **17d** enhanced antifungal activity against *A. niger* and *A. clavatus* compared with the 3-Cl substituted thiophenol **17a**. SAR studies indicated target compounds containing halogen-substituted thiophenol moiety exhibited better antifungal potency than other series.

**Figure 18:**
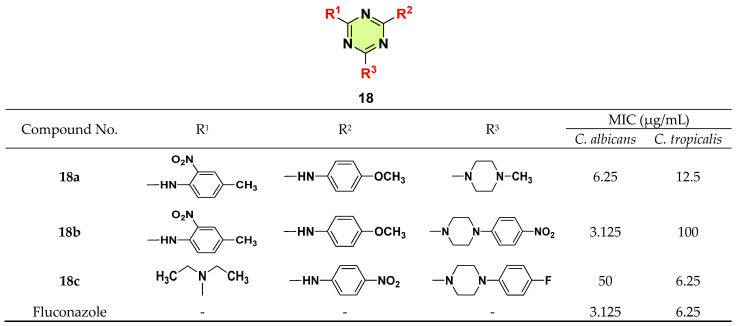
Chemical structures and antifungal activities of the s-triazine derivatives **18a**–**c**.

In a study by Singh et al. 2015, a series of 2,4,6-trisubstituted-s-triazine derivatives were synthesized and assessed for their antimicrobial activity (Figure 18) [41]. Compounds **18a**–**c** demonstrated antifungal potency comparable to fluconazole. For example, **18b** demonstrated the most significant antifungal activity against *C. albicans* (MIC = 3.125 μg/mL) and **18c** was most active against *C. tropicalis* (MIC = 6.25 μg/mL), which was equipotent to fluconazole. Replacement of the *N*-aryl piperazine group (**18b**) with *N*-methyl (**18a**) made the compounds more active against *C. tropicalis*. Nevertheless, there was no clear SAR conclusion between the structures and antifungal activity.

**Figure 19:**
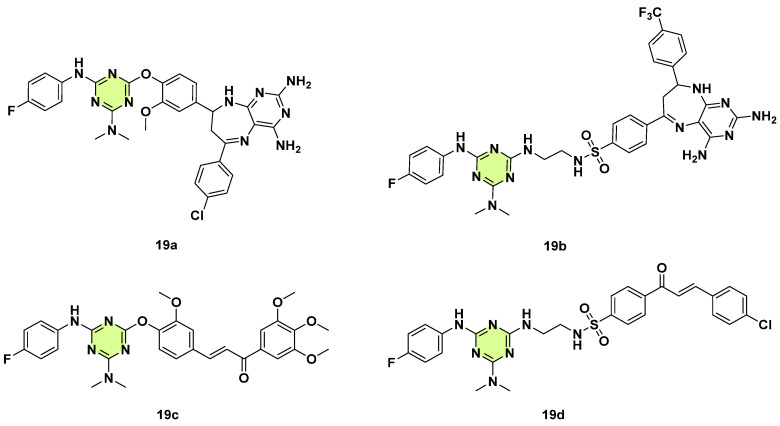
Chemical structures of the s-triazine derivatives **19a**–**d**.

A panel of s-triazine-based chalcone- and pyrimido[4,5-b][1,4]diazepine hybrids were developed by Moreno et al. in 2024 (Figure 19) [42]. The antifungal activity of these conjugates was evaluated against two yeasts *C. albicans* and *Cryptococcus neoformans*, three dermatophytes, *Microsporum gypseum*, *Trichophyton rubrum*, and *Trichophyton mentagrophytes*, and three filamentous fungi, *A. fumigatus*, *A. niger*, and *A. flavus*. Among them, the s-triazine-triazinyloxy-diazepine conjugate **19a** showed moderate antifungal activity against *T. rubrum* (MIC: 62.5 μg/mL), and s-triazine fused triazinylamino-diazepine **19b** displayed moderate potency against *T. mentagrophytes* and *A. fumigatus* (MIC: 62.5 μg/mL). Hybrid **19c** showed marginal activity against *T. rubrum* and *A. niger* (MIC: 125 μg/mL), and **19d** had the same activity against *T. mentagrophytes* with MIC = 125 µg/mL. A hemolytic assay and in silico toxicity prediction demonstrated that most of the synthesized compounds are safe. Thus, these s-triazine-based chalcone/diazepine hybrids offer an excellent framework for further optimization.

**Figure 20:**
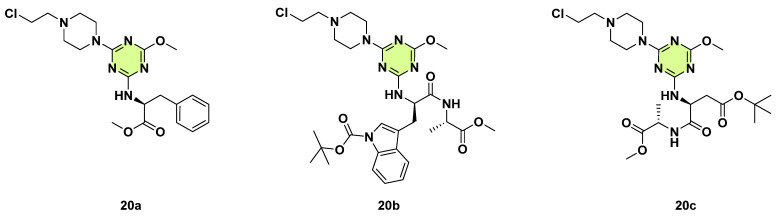
Chemical structures of the s-triazine derivatives **20a**–**c**.

In 2023, Maliszewski et al. conducted an in vitro study to investigate the antifungal potential of novel 2,4,6-trisubstituted s-triazine derivatives, which contained amino acids or short peptide chains, 2-chloroethylpiperazine, and a methoxy group (Figure 20) [43]. The study evaluated the activity against yeasts (*C. albicans*), and filamentous fungi (*A. fumigatus*, *A. flavus*, *Fusarium solani*, and *Penicillium citrinum*) using the microbroth dilution method. Antifungal agents ketoconazole and nystatin served as positive controls. All compounds were more effective against *C. albicans* than other filamentous fungi. In particular, compounds **20a**–**c**, which incorporated the -NH-PheOMe, -NH-Trp(Boc)-AlaOMe, and -NH-Asp(tBu)-AlaOMe functional groups, were more effective against *C. albicans* at a lower dose (MIC: 7.81–62.50 µg/mL) than ketoconazole and nystatin (MIC: 250 µg/mL). The studied compounds also showed broad-spectrum antibacterial effects.

**Figure 21:**
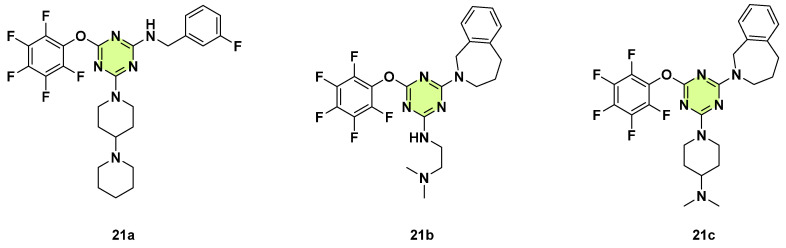
Chemical structures of the s-triazine derivatives **21a**–**c**.

Conrad et al. (2023) screened several classes of prohibitin inhibitors for antifungal activity studies (Figure 21) [44]. They identified that three melanogenin analogs **21a**–**c** containing an s-triazine ring that inhibited *C. albicans* growth at a concentration of 16.08 µg/mL, and compound **21c** completely blocked *C. albicans* growth. Compound **21c** was further selected to determine the MIC by the microbroth dilution method. Various pathogenic fungal strains were tested, including *C. albicans* SC5314, SN250, DAY185, and DAY286, *C. albicans* clinical isolates MC99 and MC102, fluconazole-resistant *C. albicans* clinical isolate 3147, *C. glabrata*, *C. tropicalis*, *C. parapsilosis*, *Candida dubliniensis*, and *S. cerevisiae*. Compound **21c** had broad spectrum antifungal activity with MICs ranging from 4–16 µg/mL. Viability analysis of *C. albicans* by flow cytometry demonstrated that **21c** had fungicidal profiles with MIC of 8–16 µg/mL. Moreover, **21c** inhibited *C. albicans* hyphal formation at sublethal concentrations (≥1 µg/mL). Although **21c** targeted the inner mitochondrial integral membrane prohibitin proteins in human cancer cells, it did not impact *C. albicans* mitochondrial activity. The MIC of **21c** in prohibitin mutant strains (*phb1* or *phb2* ∆/∆, *phb1* ∆/∆-*phb2* ∆/∆, and *phb1* ∆/∆-*phb2* ∆/∆-*phb12* ∆/∆) corresponded to the wild-type parental strain, indicating a new fungal-specific mode of action.

**Figure 22:**
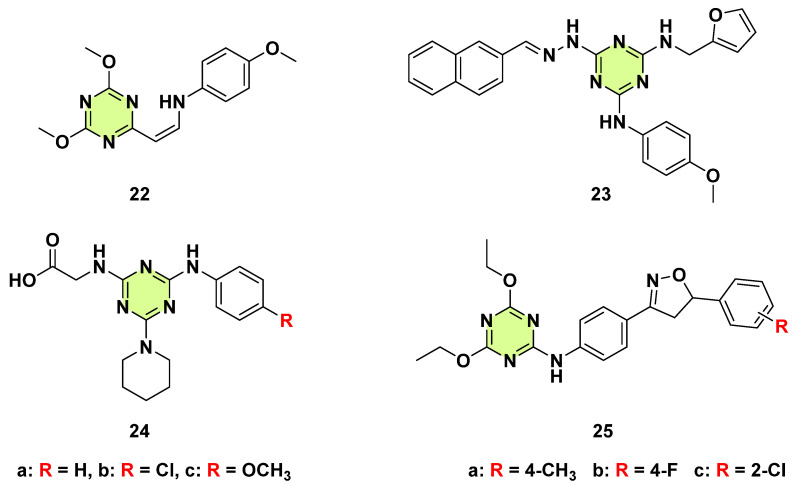
Chemical structures of the s-triazine derivatives **22**–**25**.

Mena et al. (2022) screened 90 potential biological compounds from the JUNIA chemical library to assess their antifungal effects against *C. albicans* (Figure 22) [45]. One of the s-triazine based compounds, namely (*Z*)-*N*-(2-(4,6-dimethoxy-1,3,5-triazin-2-yl)vinyl)-4-methoxyaniline (**22**), displayed rapid fungicidal activity against *C. albicans* and was also effective against fluconazole-resistant or caspofungin-resistant clinical isolated *C. albicans* strains. Confocal microscopy revealed that compound **22** could modulate the *C. albicans* cell wall by reducing the thickness of the mannan, thereby affecting *C. albicans* virulence. In the *Caenorhabditis elegans* infection model, **22** prolonged the nematodes’ survival rate and increased the expression of immune related genes, such as *lys*-1, *lys*-7, *cnc*-4, and *pmk*-1, that promote nematodes against *C. albicans* infection. Overall, this study indicates that **22** represented a promising lead compound for the treatment of *C. albicans* infections. Possible target identification and synthetic study are under investigation.

In 2018, Dong et al. carried out a virtual screening of 287,000 compounds in the Specs 3D database for identifying secreted aspartic proteases 2 (SAP2, an important virulence factor) inhibitors of *C. albicans* (Figure 22) [46]. Seven compounds had an IC_50_ value lower than 100 μM. Among them, s-triazine based compound **23** showed certain SAP2 inhibitory activity (IC_50_ = 77.18 μM). Molecular docking revealed that the triazine core was located at the central part in the active site of *C. albicans* SAP2 (PDB ID: 1EAG). Three side chains attached to the core formed π–π and hydrophobic interactions with surrounding amino acid residues. Interestingly, **23** was inactive in the antifungal assay (MIC > 64 μg/mL), which is consistent with the action mode of virulence inhibitors (SAP2 gene not affecting the in vitro growth of *C. albicans*). To evaluate the antifungal potency of **23**, a series of in vivo studies were performed. Notably, **23** exhibited potent in vivo efficacy in a mouse model of invasive candidiasis. Therefore, SAP2 inhibitors, by targeting fungal virulence factors, could be an effective way to develop new antifungal agents.

In 2020, Alhameed et al. presented the synthesis and biological assessment of 4,6-disubstituted s-triazin-2-yl amino acid derivatives (Figure 22) [47]. Among them, s-triazine with piperidine, glycine, and aniline derivatives (**24a**–**c**) showed the best inhibitory capacity at 50 µg per disc of 15 ± 0.2, 13 ± 0.1, and 14 ± 0.2 mm, respectively. The MIC and minimum fungicidal concentration (MFC) values of **24a**–**c** against *C. albicans* ranged between 34.36–37.95 µM, and 68.72–75.90 µM, respectively. The SAR showed that piperidine is the key substitution for the antifungal activity. Additionally, non-substituted aniline appeared to be more active than chlorine and methoxy-substituted compounds. Docking studies revealed that these synthesized compounds were well accommodated in the binding site of *C. albicans N*-myristoyltransferase (NMT, PDB code: 1IYL), which could be used as potential NMT inhibitors to exert antifungal activity. Interestingly, all compounds were inactive against Gram-positive and Gram-negative bacteria.

Dongre et al. (2017) synthesized a series of 4,6-diethoxy-*N*-(4-(4,5-dihydro-5-phenylisoxazol-3yl)phenyl)-1,3,5-triazin-2-amines and screened for their in vitro antifungal activities against *A. niger*, *A. flavus*, *Penicillium chrysogenum*, and *Fusarium moniliforme* by the poison plate method (Figure 22) [48]. Most of the compounds inhibited fungal growth with compounds **25a**–**c** identified as the most active.

**Figure 23:**
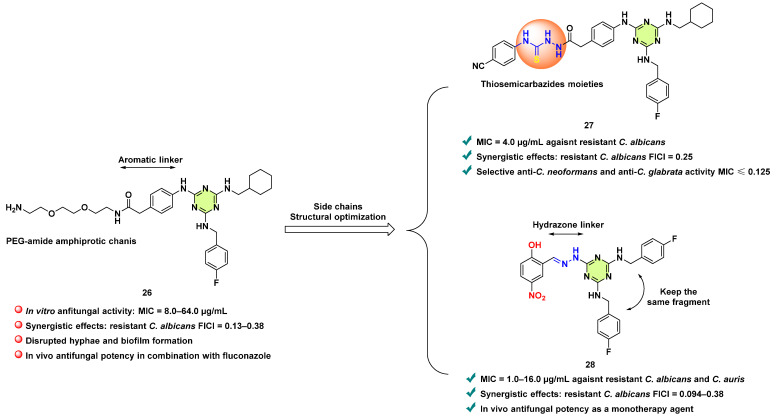
Design strategies and chemical structures of the s-triazine derivatives **26**–**28**.

Li et al. in 2019 investigated and reported the antifungal properties of a 2,4,6-triamine-substituted s-triazine derivative **26** (ENOblock) by a drug repurposing strategy (Figure 23) [49]. Compound **26** is the first reported non-substrate small-molecule inhibitor of human enolase [50]. As a homolog of human enolase, enolase 1 (Eno1) is also expressed in *C. albicans* and is essential for the growth and virulence of *C. albicans* [51]. Thus, the author first examined the antifungal activity of **26** against various fungal pathogens, including *C. albicans*, *C. neoformans*, *C. krusei*, *C. tropicalis*, *C. glabrata*, and *C. parapsilosis*. As expected, the MICs of **26** against these tested strains ranged from 8.0–64.0 µg/mL. The combination of **26** and fluconazole significantly reduced the MICs and exhibited a significant synergistic effect. Compound **26**, alone or in combination with fluconazole, showed remarkable inhibitory effects on hyphal and biofilm formation of *C. albicans* SC5314. Importantly, the combination of **26** and fluconazole showed in vivo activity against *C. albicans* SC5314 in a murine model of systemic candidiasis. The author determined **26** could directly interact with *Ca*Eno1 and inhibited the transglutaminase activity of this enzyme (IC_50_ = 12.6 µM). Taken together, **26** was identified as a novel antifungal lead for further modification.

In 2022, Xie et al. then conducted a series of structural modifications of **26** (Figure 23) [52]. They designed and synthesized forty-two novel s-triazine derivatives by replacement of the ENOblock PEG-containing side chains. Among them, the series compounds containing a thiosemicarbazide moiety exhibited excellent synergistic activity with fluconazole against fluconazole-resistant *C. albicans* (combination MIC: 0.125–2.0 μg/mL, FICI: 0.127–0.25). Of particular note, compound **27** displayed activity against resistant *C. albicans* with MIC values 4.0 μg/mL and exhibited fungal-selective inhibitory effects on *C. neoformans* (MIC ≤ 0.125–0.5 μg/mL) and *C. glabrata* (MIC ≤ 0.125 μg/mL). It was concluded that the thiosemicarbazide moiety is an important pharmacophore for generating antifungal activity.

Furthermore, Xie and colleagues, in 2024, unified two amino-substituted moieties by 4-fluorophenylmethanamine, and replaced the PEG-amide containing side chains with a hydrazone moiety (Figure 23) [53]. Therefore, several triazine hydrazone derivatives have been synthesized. Out of all derivatives, compound **28** not only showed excellent in vitro synergy in combination with fluconazole (combination MIC: 0.25–2.0 μg/mL, FICI range: 0.094–0.38) but also had direct antifungal potency against fluconazole-resistant *C. albicans* and *Candida auris* (MIC: 1.0–16.0 μg/mL). The SAR studies revealed that *ortho*-hydroxyl-substituted triazine hydrazones are the key pharmacophore. Moreover, **28** (10 mg/kg) effectively reduced the kidney burden in *C. albicans* SC5314, therefore highlighting this compound as a promising antifungal candidate.

**Figure 24:**
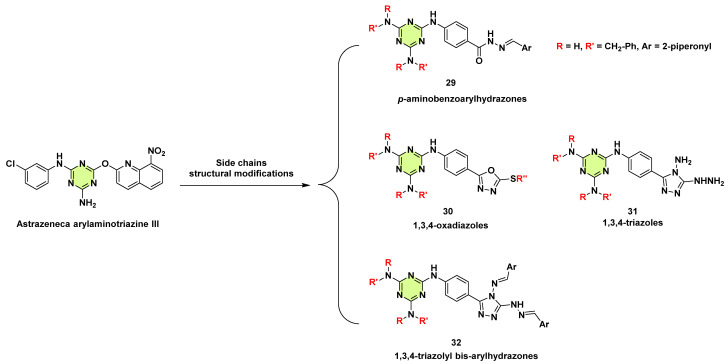
Design strategies and chemical structures of the s-triazine derivative **29**–**32**.

Haiba et al. (2019) designed and synthesized thirty-five new s-triazine derivatives based on the structure of the gyrase inhibitor Astrazeneca arylaminotriazine III, and the derivatives (**29**–**32**) were evaluated for their antibacterial and antifungal activities (Figure 24) [54]. Among them, 21 of 35 target compounds showed an inhibitory effect against *C. albicans* with MICs ranging from 25 to 100 μg/mL. The most active compound **29** displayed a lower MIC of 25 μg/mL compared to the reference clotrimazole (MIC: 12.5 μg/mL). Interestingly, it had no antibacterial activity against *Staphylococcus aureus* or *Escherichia coli*.

**Figure 25:**
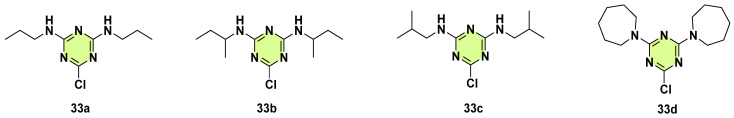
Chemical structures of the s-triazine derivatives **33a**–**d**.

In 2023, Salaković et al. investigated eight symmetrical s-triazine derivatives characterized with the same *N*-alkane or *N*-cycloalkane substituent on the N^2^ and N^4^ position and evaluated them for their in vitro antifungal activity towards *A. flavus* (Figure 25) [55]. All analyzed compounds expressed significant antifungal activity, with compounds **33a**–**c** containing an acyclic substituent, and **33d**, containing cyclic substituents, possessed the highest inhibitory activities (inhibition zone: 20.3 ± 0.6 mm). The author also carried out a comparative molecular docking to analyze the compounds’ binding affinity on the enzymes of *A. flavus*.

**Figure 26:**
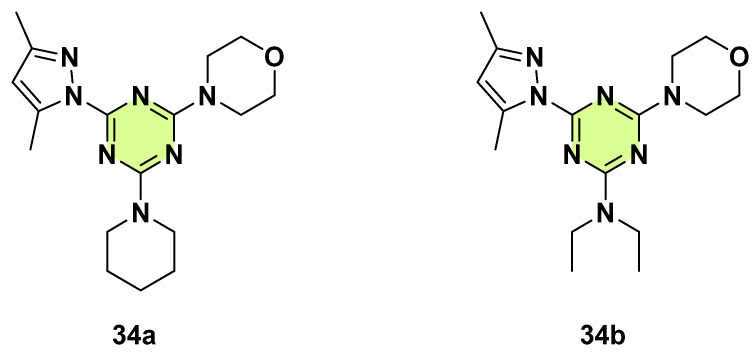
Chemical structures of the s-triazine derivatives **34a**,**b**.

Sharma et al. in 2017 reported the modification of amine-substituted s-triazine by incorporating different combinations of mono- or di-pyrazole, piperidine, benzylamine, aniline, and diethylamine moieties (Figure 26) [56]. The activity of the derivatives against *C. albicans* was tested by the agar-well diffusion method. The s-triazine bearing bis-pyrazole ring derivatives had no antifungal activity compared to mono-pyrazole. Among the mono-pyrazole compounds, the presence of the morpholine ring along with piperidine or diethylamine, like compounds **34a** (inhibition zone: 9 mm) and **34b** (inhibition zone: 8 mm), were good for anti-*C. albicans* activity.

**Figure 27:**
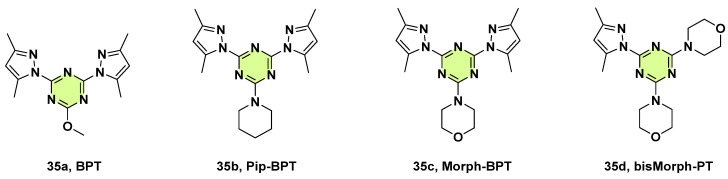
Chemical structures of the s-triazine derivatives **35a**–**d**.

Triazine derivatives with additional N or S donor atoms exhibit strong chelating abilities and provide potential binding sites for complexation with various metal ions, thereby causing considerable biological activity [57]. In 2020, Soliman et al. presented two novel zinc (II) pincer complexes, [Zn(BPT)(NO_3_)_2_] and [Zn(BPT)(H_2_O)Cl]ClO_4_, using a bis-pyrazolyl-s-triazine (**35a**, BPT) ligand (Figure 27) [58]. The ligand and its metal complexes were screened for in vitro antimicrobial activity against a panel of pathogenic strains. It was found that Zn(II) complexes exhibited broad-spectrum antimicrobial activity against the Gram positive (*Bacillus subtilis*, *Bacillus cereus*) and Gram-negative bacteria (*E.coli*, *Pseudomonas aeruginosa*, *S. aureus*), as well as the fungus *C. albicans*. Particularly, one complex, [Zn(BPT)(NO_3_)_2_], had the minimum inhibitory effect against *C. albicans* (MIC: 2.8 μmol/mL), which was superior to amoxicillin (MIC: 3.0 μmol/mL). In comparison with the related work, Refaat et al. (2022) reported two Zn(II) complexes, [Zn(BPT)(NCS)_2_] and [Zn(BPT)(Br)_2_], that showed either weak or no antifungal activity against *C. albicans* and *A. fumigatus* [59].

Using the same ligand (**35a**), Soliman et al. in 2020 and 2021 continuously developed a novel Co(II) complex, [Co(BPT)(NO_3_)_2_], and a novel Fe(III) pincer complex, [Fe(BPT)(CH_3_OH)Cl_2_], with respective MIC values of 3.2 μmol/mL and 6.2 μmol/mL against *C. albicans* [60,61]. In contrast, the Ni(II) complexes were inactive against *C. albicans* and *A. niger* [62]. In another work reported by Soliman et al. in 2020, similar Fe(III) complexes with mono- and bis-pyrazolyl s-triazine ligands (**35b**–**d**) showed good activity against *C. albicans* with MICs in the range of 18.8–37.5 µg/mL (Figure 27) [63]. In 2024, Yousri et al. synthesized Co(II), Mn(II), and Ni(II) complexes with the **35c** ligand [64]. The three studied complexes have certain inhibitory against *A. fumigatus* and *C. albicans*. It was noted that the antimicrobial activities of these metal complexes depend not only on the metal ion but also on the structure of the s-triazine ligand.

**Figure 28:**
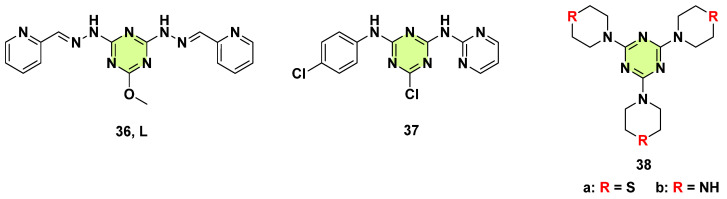
Chemical structures of the s-triazine derivatives **36**–**38**.

Soliman et al. also reported Mn(II) complexes with a new s-triazine bis-Schiff base chelating ligand (**36**, L) in 2018 (Figure 28) [65]. Antimicrobial studies showed that the [MnL(H_2_O)_2_](NO_3_)_2_ complexes are the best as antifungal and antibacterial agents. Similar work by using different s-triazine Schiff bases as ligands to form metal complexes for antifungal was reported by Fathalla et al. in 2023 [66,67,68]. Al-Khodir et al. (2019) assessed the antimicrobial and anticancer activities of Ru(III) and Se(IV) complexes containing an s-triazine chelating ligand [69]. The results showed that all Se(IV) complexes have a higher activity against *A. flavus* and moderate activity against *C. albicans* compared to Ru(III) complexes and amphotericin B. The 6-chloro-*N*^2^-(4-chlorophenyl)-*N*^4^-(pyrimidin-2-yl)-1,3,5-triazine-2,4-diamine) ligand (**37**, Figure 28) with a Se(IV) complex introduced the most promising efficiency.

Martins et al. (2019) synthesized 2,4,6-tris(thiomorpholine)-1,3,5-triazine (**38a**, TMT), 2,4,6-tris(piperazine)-1,3,5-triazine (**38b**, PIPT), and their Sb(III) and Bi(III) complexes (Figure 28) [70]. The results from antimicrobial assays showed that the Sb(III) complexes, ([SbCl_3_(TMT)], [Sb_3_Cl_9_(TMT)_2_], and [Sb_2_Cl_6_(PIPT)].4H_2_O), had antifungal activity against *S. aureus*, *C. albicans*, *C. tropicalis*, and *C. krusei* with MICs in the range of 512–1024 µg/mL. Additionally, two free ligands (TMT, PIPT) and SbCl_3_ did not inhibit the growth of the evaluated microorganisms, suggesting that coordination of metal ions through s-triazine based ligands is a good strategy for the development of new antimicrobial agents.

**Figure 29:**
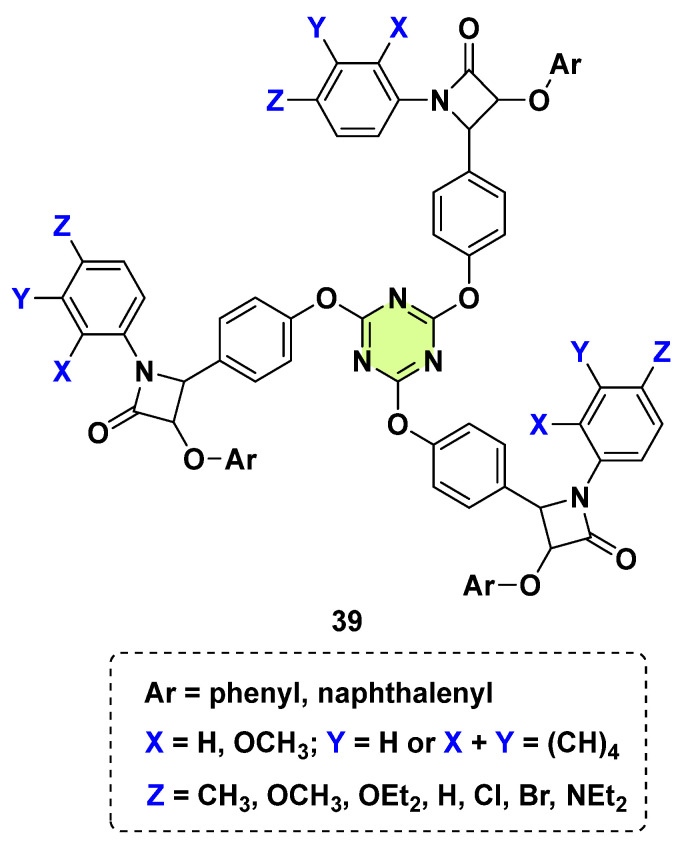
Chemical structures of the s-triazine derivatives **39**.

Due to the branching capabilities of the s-triazine nucleus, many mono-, bi-, and tri-substituted compounds can be prepared by controlling the reaction conditions. In this regard, Bashiri et al. in 2020 synthesized a collection of tris-β-lactam 1,3,5-triazine hybrids (**39**) and investigated their potential biological activities (Figure 29) [71]. Although some hybrid molecules showed antiproliferative, antibacterial, and antioxidant properties, they were inactive against the two tested fungi (*C. albicans* and *A. fumigates*). Vembu et al. (2016) designed and synthesized a series of 2,4,6-tris(tetrazol-1-yl-(4-phenyl(3-arylpropene-1-on-1-yl))-1,3,5-triazines (dendrimers) [72]. Some compounds exhibited moderate fungicidal activities and were more active against *C. albicans*, *S. cerevisiae*, and *Aspergillus* spp. than fluconazole.

**Figure 30:**
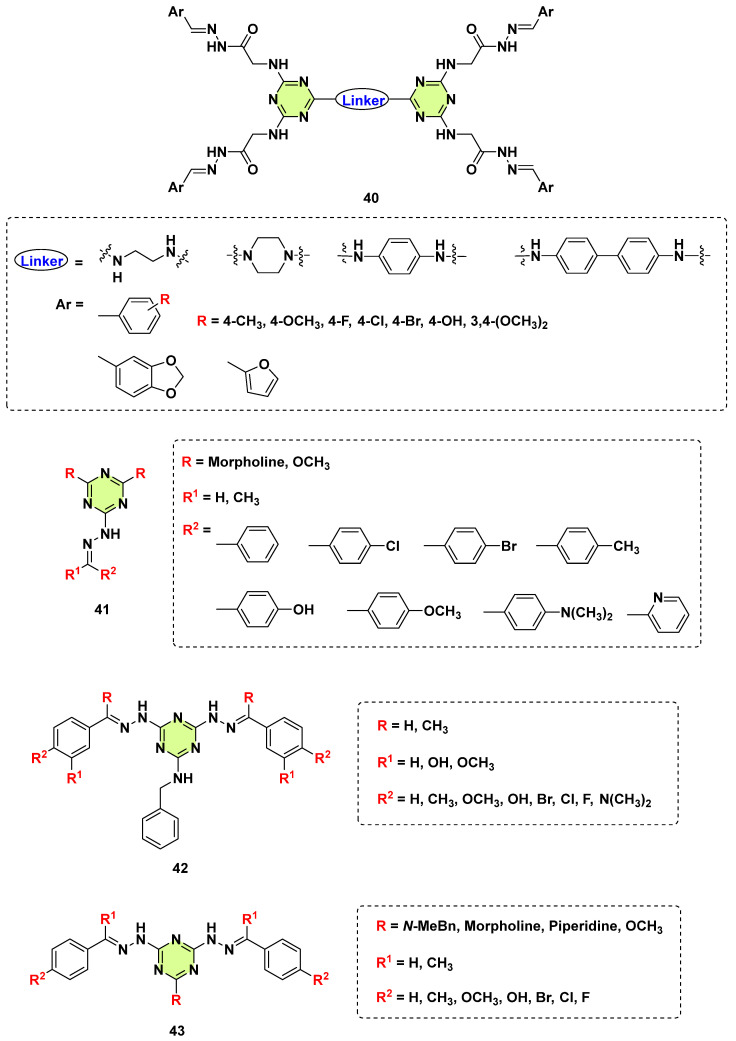
Chemical structures of the s-triazine derivatives **40**–**43**.

It is well known that Schiff bases (−NH−N=CH−) have numerous biological activities. In 2018, Ramadan et al. synthesized and reported a novel class of dimeric s-triazine hydrazide derivatives (**40**) using 1,2-diaminoethane, 1,4-diaminocycloalkane, 1,4-diaminobenzene, and 1,1’-biphenyl-4,4’-diamine as linkers (Figure 30) [73]. The synthesized compounds were investigated for their in vitro antimicrobial activity. Unfortunately, none of the dimeric s-triazine hydrazide showed anti-*C. albicans* activity. Also, Al-Rasheed et al. synthesized series of s-triazine based Schiff bases derivatives (**41**–**43**) (Figure 27) [74,75]. Some target compounds exhibited good antibacterial activity; however, none of them showed a specific effect against the tested fungi.

**Figure 31:**
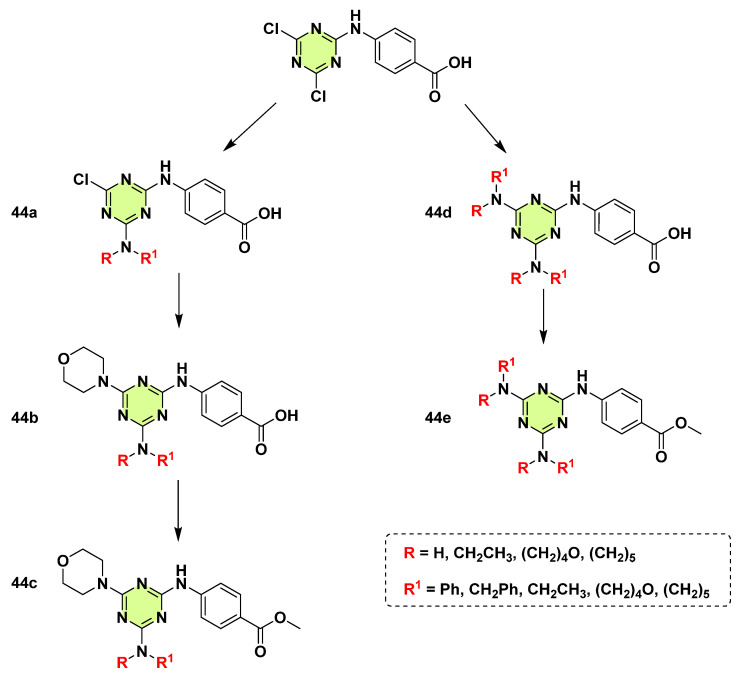
Chemical structures of the s-triazine derivatives **44a**–**e**.

Al-Zaydi et al. (2017) synthesized a series of s-triazine based aminobenzoic acids and their methyl ester analogs (**44a**–**e**) (Figure 31) [76]. In vitro MIC antifungal results showed all the tested compounds have no inhibition activity against *C. albicans* (MIC > 200 μg/mL).

## 4. Discussion

Presently, limited anti-IFIs drugs are available in clinical settings. Of those available, there are three predominant classes: azoles, polyenes, and echinocandins. Azoles, including triazole and tetrazole, are fungal lanosterol 14α-demethylase (CYP51, encoded by the *erg11*) inhibitors, which disrupt cell membrane integrity and thereby block fungal cell growth. Azoles are widely used due to their broad-spectrum activity, particularly against *Candida*, *Cryptococcus*, and *Aspergillus*. However, azoles also inhibit cytochrome P450 enzymes and are fungistatic, which leads to hepatotoxicity, drug–drug interactions, and severe drug resistance. Amphotericin B (AmB) is a clinically used polyene that binds to the cell membrane ergosterol, leading to cell death. Although AmB is the earliest-used drug and remains the “gold standard” for treating IFIs, it has severe toxicity due to the similarity between ergosterol and cholesterol in mammalian cell membranes. This side effect and low oral bioavailability curb its clinical application. Echinocandins inhibit fungal growth by targeting β-(1,3)-glucan synthesis, which is an essential component of the fungal cell wall. These agents display excellent fungicidal activity against various *Candida* species and are commended as first-line drugs in patients suffering from invasive *Candida* infection. The primary drawbacks of echinocandins include poor oral bioavailability, ineffectiveness against *Cryptococcus* species, and emerging resistance in non-*C. albicans*. Remarkably, each antifungal drug class has significant therapeutic limitations, ranging from toxicity to drug resistance. Therefore, discovering novel antifungal agents with new chemical scaffolds is urgently needed.

This review covers the advancement of s-triazine and its derivatives with potential antifungal activities against fungal strains, and SARs are also analyzed briefly. As an essential class of the heterocyclic compound, the triazine ring is a six-membered benzene structure with three carbons replaced by nitrogen. Among the three isomeric forms of triazine, s-triazine occupies a key position in medicinal chemistry because of its wide applications and high biological activities. As one of the primary starting materials, cyanuric chloride can afford numerous s-triazine derivatives (mono-substituted, di-substituted, and tri-substituted), taking advantage of its low cost and easy manipulation via multiple nucleophilic substitution reaction. This lies in further conjugating with active pharmacophores of the s-triazine core through molecular hybridization. Some general conclusions could be obtained based on the SARs from the above-mentioned s-triazine derivatives:

- Substituents on the s-triazine core with tetrazole, pyridine, pyrimidine, morpholine, piperazine, piperidine, coumarin, chalcone, and quinoline are favored for improving their antifungal activity against *Candida*, *Cryptococcus*, and *Aspergillus* species.
- Halogen atoms, especially fluorine, chlorine, and bromine, are present in the s-triazine derivatives’ chemical structures, which suggests the significant importance of halogen bonding in interactions with corresponding targets.
- Tri-substituted s-triazine derivatives are generally more active than di-substituted and mono-substituted, possibly due to the introduction of more pharmacophore fragments.

On the other hand, triazines are more often used in studies of antibacterial, antitumor, antiviral, and other fields. Due to the lack of in-depth antifungal mechanisms and target studies, most of the s-triazine derivatives mentioned in the text are obtained based on phenotypic screening or via medicinal chemistry driven SARs studies, not by structure-based drug design or target-based drug discovery. Therefore, further pharmacology experiments are warranted to investigate the corresponding mechanism/targets of s-triazine antifungal compounds. Moreover, applying s-triazine derivatives in clinical practice still faces significant challenges. Promising compounds need further evaluation, including a susceptibility test against other fungal species, safety evaluation (e.g., hemolytic activity or cytotoxicity against mammalian cell lines), unraveling mechanisms of action (e.g., enzyme inhibition, cell membrane/wall disruption), and a deeper understanding of pharmacokinetic/pharmacodynamic (PK/PD) properties, as well as in vivo efficacy studies.

## 5. Conclusions

Fungal infections have posed a heavy burden on the world health system. The current treatment of fungal disease is compounded by its efficacy, toxic side effects, bioavailability, and emergence as a drug-resistant fungi. s-Triazine displays a broad spectrum of pharmacological activities, acting as a versatile scaffold for drug design and development. Several s-triazine derivatives have recently been reported for their antifungal activities, and some exhibited promising in vitro and in vivo potency against drug-sensitive and drug-resistant fungal pathogens. This review covers the recent advances of s-triazine compounds as potential antifungal agents, and the effect of different substituents installed on the s-triazine core is also discussed. More synthesis and SAR studies will bring new perspectives for further optimization of s-triazine compounds.

## 6. Future Prospects

The synthesis of s-triazine derivatives will continue to be a research focus. Nevertheless, it should be noted that most of the above-mentioned studies lack comprehensive evaluation or do not determine the exact antifungal mechanism of the s-triazine derivatives. There still remains adequate room for exploring s-triazine compounds and their underlying antifungal activity. For example, by carrying out structure-based drug design (SBDD), computer-aided drug design (CADD), and even the proteolysis-targeting chimeras (PROTAC) technique, chemists can further construct multifunctional s-triazine derivatives that possess multi-targeting, membrane-targeting, protein–protein interactions, inhibition, anti-virulence, and/or anti-drug efflux characteristics. As such, s-triazine/s-triazine derivatives are promising chemical entities for antifungal activity screening, and sufficient effort should be devoted to exploring their potential. Based on more systematic studies, novel s-triazine antifungal candidates with a broad-spectrum, higher activities, and a lower toxicity are worth expecting in future drug discovery.

## Data Availability

Not applicable.
